# Changes in serum Interleukin-33 concentration before and after treatment with pegylated interferon alfa-2a plus ribavirin in patients with chronic hepatitis C genotype 1b infection

**DOI:** 10.5812/hepatmon.7611

**Published:** 2012-12-23

**Authors:** Bruno Cacopardo, Marilia Rita Pinzone, Filippo Palermo, Giuseppe Nunnari

**Affiliations:** 1 Department of Clinical and Molecular Biomedicine, Division of Infectious Diseases, University of Catania, Catania, Italy; 2 Department of Clinical and Molecular Biomedicine, Division of Infectious Diseases University of Catania, Catania, Italy; 3 Department of Microbiology and Immunology, Jefferson Medical College, Thomas Jefferson University, Philadelphia, USA

**Keywords:** Hepatitis, Chronic, Disease Progression, Fibrosis, IL33 Protein, Humans, Interferons, Hepatitis C

## Abstract

**Background:**

IL-33 is a novel member of the IL-1 family, which has been shown to play an important role in T helper 2 (Th2)-associated immune responses. Recent studies have suggested a possible role for IL-33 in the pathogenesis of liver damage during acute and chronic hepatitis; furthermore, IL-33 may be involved in the development and progression of liver fibrosis.

**Objectives:**

To evaluate serum IL-33 levels in a group of patients with chronic hepatitis C (CHC) genotype 1b at enrolment and after a course of pegylated (PEG)-IFN plus ribavirin.

**Patients and Methods:**

60 patients with chronic hepatitis C (CHC) and 65 healthy controls were examined and compared for serum IL-33 levels by ELISA. All CHC patients were submitted to liver biopsy either before starting antiviral treatment or during post-treatment follow up. We evaluated whether post-treatment IL-33 concentration was associated with histologic outcome as well as with virologic response to therapy.

**Results:**

Serum IL-33 levels were significantly higher among CHC patients in comparison with healthy controls. IL-33 concentration was lower among patients with a METAVIR fibrosis score F1-F2, compared with those having a more advanced liver disease (METAVIR stage F3-F4). In addition, sustained virologic response (SVR) was associated with a significant drop in IL-33 levels, whereas no changes were found among relapsers and nonresponders. Analogously, patients experiencing liver histologic improvement after antiviral therapy had lower post-treatment IL-33 levels in comparison with baseline values. Contrarily, no variations were detected among subjects with worsened or stable histologic features.

**Conclusions:**

IL-33 may represent a new and easy-to-detect biomarker for the diagnosis of liver damage in CHC patients, as it appears to be modulated in parallel with biochemical and histologic parameters, such as ALT levels and liver fibrosis. Furthermore, considering that serum IL-33 concentration was significantly reduced following a successful course of antiviral treatment, this cytokine may also represent a sensitive indicator of SVR.

## 1. Background

Interleukin (IL)-33 is among the most recent cytokines to be identified (1). It belongs to the IL-1 family and is also known as nuclear factor of high endothelial venules (NF-HEV)(2) and DVS 27 (3). IL-33 plays an important role in T helper 2 (Th2)-associated immune responses: in fact, IL-33 expression has been shown to drive in vitro the production of Th2 cytokines from polarized Th2 cells and to induce in vivo the expression of IL-4, IL-5, IL-10 and IL-13 (1). IL-33 is a ligand for the IL-1 receptor family member suppression of tumorigenicity 2 (ST2), whose signal pathway involves Myeloid differentiation primary response gene 88 (MyD88) and Nuclear Factor-KappaB (NF-κB) (1, 4). IL-33 over-expression has been associated in mice with allergic airway diseases and autoimmune diseases (5-11). It acts as a proinflammatory and chemoattractant cytokine, enhancing massive cellular infiltration in the sites where inflammation takes place (12-14). Recent reports hypothesized IL-33 to be involved in the pathogenesis of liver damage during acute and chronic hepatitis (15-17). In addition, IL-33 levels have been positively correlated with liver fibrosis in mice and humans (15, 18). A recent Chinese study reported IL-33 over-expression to be associated with the development and progression of hepatitis C virus (HCV)-related liver fibrosis (15). Furthermore, the authors found a tight connection between serum alanine aminotransferase (ALT) and IL-33 levels, as well as a significant decrease in serum IL-33 concentration among patients achieving a drop in HCV viraemia during antiviral treatment with Interferon (IFN).

## 2. Objectives

In the present study, we measured serum IL-33 levels in a group of patients affected with chronic hepatitis C (CHC) at enrolment and after a course of pegylated (PEG)-IFN plus ribavirin. We also evaluated whether post-treatment serum IL-33 variations could be related either to the virologic or to histologic response to treatment.

## 3. Patients and Methods

### 3.1. Study Population

Between January and June 2010, 60 consecutive patients aged 18 years or older, followed at the Outpatient Clinic of Infectious Diseases (Garibaldi Nesima Hospital, University of Catania) for previously untreated chronic hepatitis C (CHC), were enrolled for cytokine detection. All 60 patients had a positive HCV antibody test and were HCV RNA positive according to a real-time polymerase-chain reaction assay (COBAS AmpliPrep/COBAS TaqMan HCV Test). All were affected with genotype 1b (by Inno LiPA HCV II, Innogenetics) infection. Forty out of 60 (66%) had serum ALT elevation (Hyper-ALTs), as assessed by alanine aminotransferase (ALT) value exceeding the upper limit of normal at least once during the previous 12 months. Contrarily, 20 patients had persistently normal ALT (PNALTs), as defined by normal ALT value on previous tests conducted every three months for a minimum of 18 months; in the laboratory of our University Hospital, the upper limit of normal ALT value was 40 IU/L. In all 60 cases liver biopsy specimens had been taken in the previous six months for hystopathological examination. Specimens had been formalin-fixed, paraffin embedded and then sectioned and stained (hematoxylin and eosin) by a single pathologist using the METAVIR histologic activity index score for grading and staging of chronic hepatitis (19). All 60 patients had contemporarily adhered to an antiviral therapy multicentric protocol, scheduling administration of PEG-IFN α-2a plus a fixed dose of oral ribavirin. To be included in the protocol for antiviral treatment, patients underwent strict selection criteria: patients with decompensated liver cirrhosis or serological evidence of HBV or HIV-1 infection were excluded, as well as patients with serum creatinine level more than 1.5 times the upper normal limit and those with absolute neutrophil count lower than 1500/µl, platelet count lower than 80.000/µl and hemoglobin level lower than 12 g/dl. Organ transplant recipients, individuals with severe cardiac disease, individuals with a history of severe psychiatric disease, individuals with evidence of drug or alcohol abuse within the previous years and individuals with other serious systemic diseases were ineligible for the study. Pregnant or lactating women were also excluded. A group of 65 healthy individuals with no history of liver disease, no serological evidence of viral hepatitis infection or chronic exposure to drugs or alcohol was enrolled as a control group from the non-medical staff working at our institution (nurses, technical and administrative employers). This study was conducted in accordance with the declaration of Helsinki; written informed consent was obtained from each subject at enrolment. Table 1 compares baseline demographic, biochemical, virologic and histologic characteristics of CHC patients and healthy controls

**Table 1:** Baseline Characteristics of the Study Population, Data Are Expressed as Mean ± Standard Deviation, Except Where Otherwise Noted

Characteristics	Patients with chronic hepatitis C (CHC)	Healthy Controls
**Subjects enrolled, No.**	60	65
**Age, y, Mean ± SD **	47.4 ± 9	48.3 ± 13
**BMI, kg/m^2^, Mean ± SD**	26.3 ± 2.1	27.7 ± 3.1
**Gender: Male/Female, No.**	39/21	37/28
**ALT, IU/L, Mean ± SD**	79.4 ± 18 a	12.6 ± 8 a
**HCV RNA, IU/ml × 10^3^, Mean ± SD**	516 ± 160	-
**Genotype 1b, No.**	60	-
**METAVIR fibrosis stage**		
F1, No.	13	-
F2, No.	20	-
F3, No.	20	-
F4, No.	7	-
**METAVIR activity grade**		;
A0, No.	9	-
A1, No.	13	-
A2, No.	22	-
A3, No.	16	-

^a^Patients with CHC vs. Healthy Controls: P < 0.01

### 3.2. Antiviral Treatment with PEG-IFN Plus Ribavirin

Before starting treatment, abdominal ultrasound and esophagogastroscopy were performed in all cases to exclude the presence of portal vein caliber dilation and the presence of esophageal or gastric varices. In June 2010, all previously untreated CHC patients started a course of treatment with PEG-IFN α-2a at a weekly dose of 180 µg plus a fixed dose of ribavirin (1000 mg/day orally). Treatment was scheduled in all cases for as long as 48 weeks. In June 2012, patients completed the one-year post-treatment follow up. Patients were submitted to direct clinical evaluation at weeks one, two, three and four and then every four weeks during treatment. Serum HCV RNA measurements were performed at weeks 4, 12, 24 and 48 during therapy and thereafter at week 24 and 48 after the end of therapy. Adverse events to therapy were evaluated with the use of World Health Organization (WHO) grades (20). For safety reasons we decided to discontinue therapy in case of grade 3 or 4 adverse events. The primary efficacy endpoint of antiviral treatment was sustained virologic response (SVR), defined as undetectable serum HCV RNA at the end of our 48-week post-treatment follow up. Patients were considered nonresponders if serum HCV RNA declined less than 2 log10 at week 12 of therapy or as an alternative in case of detectable HCV RNA at week 24: in these cases treatment was interrupted. Patients were also defined as relapsers when HCV RNA was undetectable by the end of treatment but became detectable any time during post-treatment follow up. In all cases who received antiviral therapy, a liver biopsy was repeated 40 ± 8 weeks following stopping treatment.

### 3.3. Measurement of Serum IL-33 Levels by Enzyme Linked Immunosorbent Assay (ELISA)

Peripheral blood samples were obtained from each subject and sera were stored at -80°C until IL-33 evaluation. Serum IL-33 levels were measured at enrolment and in conjunction with the repetition of liver biopsy (about 40 weeks after completing antiviral therapy). IL-33 was detected by Human IL-33 ELISA Kit (GenWay Biotech, Inc. 6777 Nancy Ridge Drive San Diego, CA, USA), according to the manufacturer’s instructions. The range of detection of the test was between 0.7 and 500 ng/ml.

### 3.4. Statistical Analysis

Most data were expressed as mean ± standard deviation (SD). Serum HCV RNA values were all expressed as UI/mL and logarithmically transformed to normalize their distribution. Unpaired Student’s t test was used to compare healthy controls’ versus pre-treatment patients’ IL-33 values, Hyper-ALTs versus PNALTs and F1-2 versus F3-4 histological staging groups. Contrarily, pre-treatment versus post-therapy results were compared by paired t test. Results of statistical comparison included mean difference and 95% confidence interval

## 4. Results

### 4.1. Outcome of Antiviral Treatment

Eight out of 60 patients (13.3%) discontinued PEG-IFN plus ribavirin treatment at week 12 because of clear-cut nonresponse. In six patients (10%) discontinuation of therapy within the first 24 weeks was due to development of grade 3-4 WHO side effects. Thus, on an intention-to-treat basis, 14 out of 60 patients (23.3%) were considered as nonresponders whereas 20 patients (33.3%) relapsed during post-treatment follow up. Comprehensively, 26 of 60 patients (43.3%) experienced SVR. Histologic improvement, defined as a reduction of at least one point score of the METAVIR fibrosis score, was observed in 50% of patients receiving PEG-IFN plus ribavirin: in detail, it was found in all 26 subjects exhibiting SVR and in 4 relapsers. Contrarily, nine patients (15%), including eight nonresponders and one relapser, developed worsening histologic fibrosis and 21 subjects (35%), including 15 relapsers and 6 nonresponders, had no histologic modifications.

### 4.2. Evaluation of Serum IL-33 Levels

**Figure 1:**
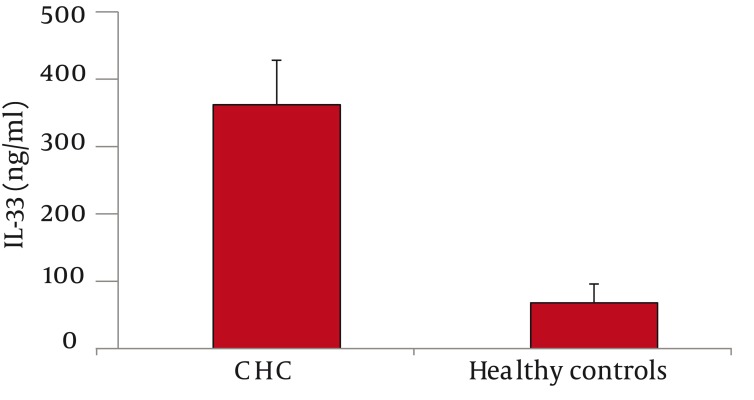
Serum IL-33 Levels among Patients with Chronic Hepatitis C (CHC) and Healthy Controls IL-33 Concentration was Significantly Higher (362 ± 66 vs. 68 ± 28 ng/ml, P < 0.001) in the CHC Cohort

**Figure 2:**
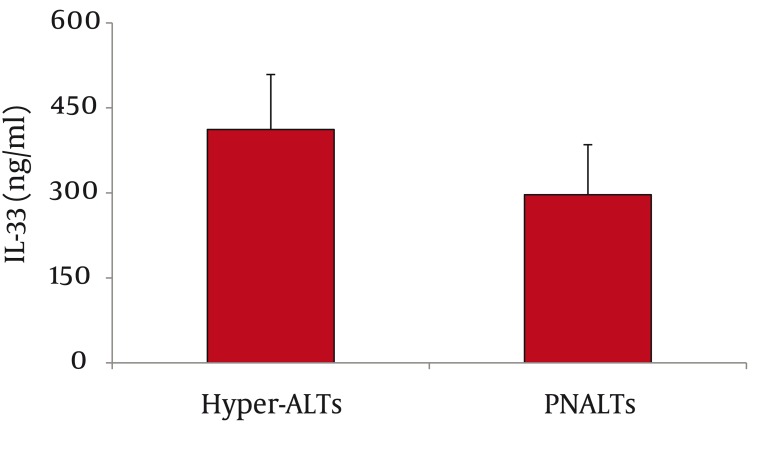
Serum IL-33 Levels among Patients with Persistently Normal Alanine Aminotransferase (PNALTs) and Patients with Elevated ALT (Hyper-ALTs). PNALTs had Significantly Lower IL-33 Levels than Hyper-ALTs (297 ± 88 vs. 412 ± 97 ng/ml, P < 0.01)

**Figure 3:**
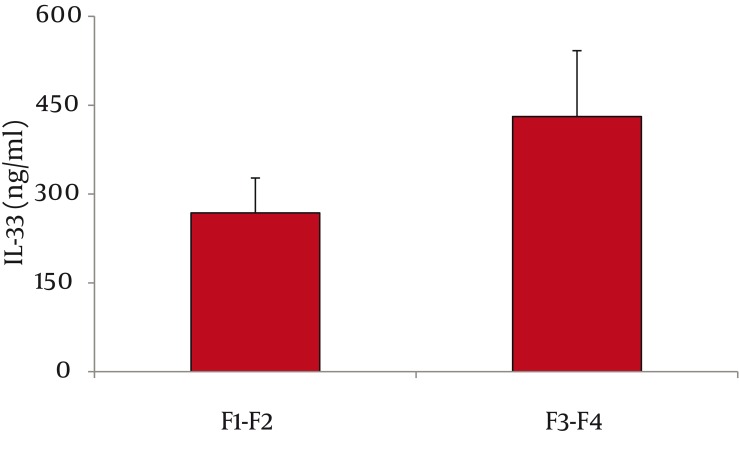
Serum IL-33 Levels and METAVIR Stage Patients with METAVIR Fibrosis Score F3-F4 had Significantly Higher IL-33 Values than Subjects with METAVIR Stage F1-F2 (431 ± 111 vs. 268 ± 59 ng/ml, P < 0.01)

As for serum IL-33 levels, we found that 60 patients with CHC had significantly higher IL-33 concentration than 65 healthy controls (362 ± 66 vs. 68 ±28 ng/ml, P < 0.001; mean difference -294, Confidence Interval (C.I.) 95% 311.7-276.3) (Figure 1). In addition, IL-33 levels were significantly lower among PNALTs in comparison with Hyper-ALTs (297 ± 88 vs. 412 ± 97 ng/ml, P < 0.01; mean difference 115; C.I. 95% 63.4-166.6) (Figure 2). Moreover, patients exhibiting a METAVIR fibrosis score of F1-F2 had a significantly lower IL-33 concentration than patients with a more advanced METAVIR fibrosis score (F3-F4) (268 ± 59 vs 431 ± 111 ng/ml, P < 0.01; mean difference 163, C.I. 95% 118.2-207.8) (Figure 3). As for antiviral therapy, overall pre-treatment IL-33 concentration was significantly higher than overall post-treatment value (362 ± 66 vs. 191 ± 39 ng/ml, P < 0.01, mean difference -171; C.I. 95% -180.6 to -131.40). Among patients who achieved SVR, post-treatment IL-33 levels considerably dropped when compared with pre-treatment values (from 402 ± 119 to 96 ± 37 ng/ml, P < 0.001; mean difference -306; C.I. 95% -325.1 to – 237.9). On the contrary, we did not find significant variations of IL-33 concentration among relapsers (371.1 ± 96 ng/ml vs. 313.0 ± 108 ng/ml; P = 0.081, mean difference -58; C.I. 95% -120.4 to 5.4) and nonresponders (318 ± 79 vs. 301 ± 84 ng/ml; P = 0.586, mean difference -17; C.I. 95% -73.3 to 40.3) (Figure 4, panel a). Analogously, patients who obtained a post-treatment improvement of liver histology also showed a significant reduction of serum IL-33 levels (from 388 ± 101 to 101 ± 56 ng/ml, P < 0.001; mean difference -388.0; C.I. 95% -508.4 to –222.8), whereas no cytokine changes were found among subjects whose histologic specimens were unmodified (306 ± 70 vs. 289 ± 91 ng/ml; P = 0.501, mean difference -17; C.I. 95% -66.3 to 31.6) or worsened (299 ± 84 vs. 290 ± 76 ng/ml; P = 0.724, mean difference -9; C.I. 95% -56.3 to 40.2) following therapy (Figure 4, panel b).

**Figure 4a:**
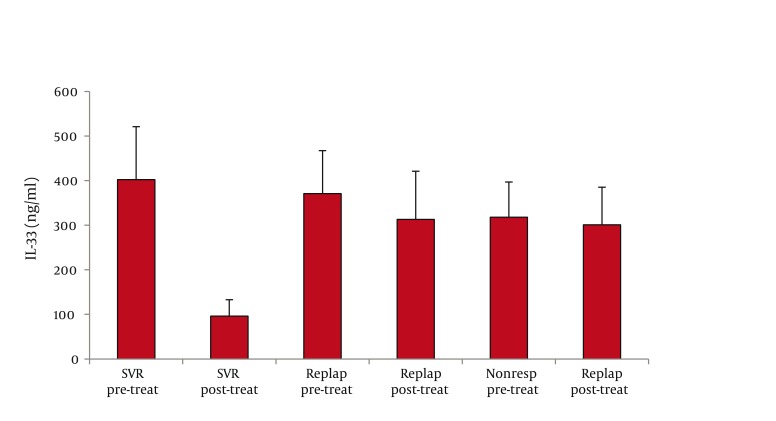
(Panel a) Evaluation of IL-33 Levels before (pre-treat) and after (post-treat) Antiviral Treatment with Pegylated Interferon plus Ribavirin among Subjects who Achieved Sustained Virologic Response (SVR), Relapsers and Nonresponders The Three Groups Had Similar IL-33 Baseline Values; a Significant Drop in IL-33 Concentration Was Found only among Patients Achieving SVR (P < 0.001)

**Figure 4b:**
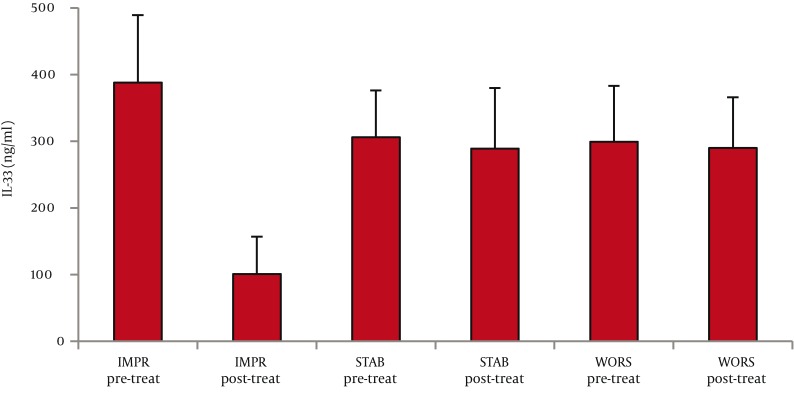
Panel b) Re-Evaluation of IL-33 Levels after Antiviral Treatment with Pegylated Interferon Plus Ribavirin among the Following Groups of Patients Patients Experiencing Histologic Improvement, Patients with no Significant Modifications of Histology and Patients with Histologic Worsening. IL-33 Concentration before Treatment was Similar in the Three Groups; a Significant Reduction of IL-33 Levels Was Found only among Subjects Experiencing Histologic Improvement after Antiviral Therapy (P < 0.001)

## 5. Discussion

IL-33 is a multifunctional cytokine, which is able to activate NF-kB signal pathway and to promote Th2 immune responses (1, 4). IL-33 has been shown to participate in the acute-phase response (12-14) and to be involved in the pathogenesis of several diseases, including allergic respiratory diseases, rheumatoid arthritis and systemic lupus erythematosus (5-11). The role of IL-33 in the development of liver damage is complex and only partially defined. On the one hand, IL-33 seems to act as an “alarmin cytokine” (1), an endogenous signal of tissue damage. IL-33 has been reported to be released from necrotic cells after viral infections; IL-33 activates in turn CD8 + T cells (CTLs), to promote virus control (2). This is a new, interesting mechanism, whereby non-hematopoietic cells may mobilize CTL response to infection, through alarmins. In addition, Natural killer (NK) T cells have been shown by Arshad et al. (17) to induce IL-33 production in hepatocytes during Concanavalin-A (Con-A) induced acute hepatitis; IL-33, released from damaged cells, may promote in turn the initiation of healing responses by engaging ST2 (1). Volarevic et al. (16) showed that IL-33/ST2 axis downregulated Con-A induced hepatitis, as supported by the evidence of more severe liver damage among ST2-deficient mice; in addition, IL-33 was reported to suppress the activation of caspase-3 and to enhance the expression of anti-apoptotic B-cell CLL/lymphoma 2 (Bcl-2) in the liver. On the other hand, IL-33 has been suggested to play a role in the development of hepatic fibrosis. Marvie et al (18) found that IL-33 mRNA, as well as IL-33 protein, was overproduced in human fibrotic liver in comparison with normal liver, with the major source of IL-33 represented by activated hepatic stellate cells (HSCs). Wang et al. (15) observed elevated serum IL-33 levels among patients affected with CHC in comparison with healthy controls; furthermore, the authors found a significant correlation between IL-33 and ALT concentration. Subcutaneous administration of 500 million units of a short-acting IFN produced a sudden and significant reduction of serum IL-33 levels. We analyzed serum IL-33 concentration in a small cohort of patients affected with biopsy-proven CHC. In comparison with the paper of Wang et al. (15), our study was further enforced by a double histologic evaluation, which was performed prior starting antiviral therapy as well as during post-treatment follow up. Our results are in keeping with those of Wang et al., since we also found higher serum IL-33 levels in CHC patients than controls and, within the CHC cohort, higher IL-33 concentration among Hyper-ALTs. In addition, as far as we know, the present study is the first to demonstrate that IL-33 levels are higher among CHC patients with more advanced fibrosis (METAVIR stage F3-F4). It could be hypothesized that serum IL-33 may work as a new and easy-to-detect biomarker for the diagnosis of liver damage in CHC patients; in fact, its levels seem to be modulated in parallel with biochemical and histologic parameters. Of importance, a successful course of antiviral treatment was able to induce a concomitant decrease in serum IL-33 concentration, so that this cytokine may represent a sensitive indicator of SVR. Nevertheless, it is still unclear whether raised IL-33 levels represent the cause or rather a consequence of the progression of HCV-related liver disease. Our study is limited by its small size: further large-scale studies are required to better characterize the role of this cytokine throughout the course of a chronic and progressive disease such as CHC.
